# Relapse Prevention Therapy for Problem Gaming or Internet Gaming Disorder in Swedish Child and Youth Psychiatric Clinics: Protocol for a Randomized Controlled Trial

**DOI:** 10.2196/44318

**Published:** 2023-01-05

**Authors:** Sabina Kapetanovic, Sevtap Gurdal, Isak Einarsson, Marie Werner, Frida André, Anders Håkansson, Emma Claesdotter-Knutsson

**Affiliations:** 1 Department of Social and Behavioral Sciences University West Trollhättan Sweden; 2 Department of Psychology Stockholm University Stockholm Sweden; 3 Child and Adolescent Psychiatry Outpatient Clinic Region Skåne Malmö Sweden; 4 Child and Adolescent Psychiatry Outpatient Clinic Region Skåne Lund Sweden; 5 Department of Clinical Sciences Faculty of Medicine Lund University Lund Sweden; 6 Malmö Addiction Center and Competence Center Addiction Region Skåne Malmö Sweden

**Keywords:** problem gaming, internet gaming disorder, parent-child relationship, randomized controlled trial, relapse prevention, psychiatry, psychology, treatment

## Abstract

**Background:**

Although gaming is a common arena where children socialize, an increasing number of children are exhibiting signs of problem gaming or internet gaming disorder. An important factor to the development of problem gaming is parent-child relationships. A cognitive behavioral therapy–based form of treatment, labeled relapse prevention, has been developed as a treatment for child and adolescent problem gaming or internet gaming disorder. However, no study has evaluated the effect of this treatment among Swedish children and youth nor the role of the parent-child relationships in this treatment.

**Objective:**

This study aims (1) to evaluate a relapse prevention treatment for patients showing signs of problem gaming or internet gaming disorder recruited from child and youth psychiatric clinics and (2) to test whether the quality of parent-child relationships plays a role in the effect of relapse prevention treatment and vice versa—whether the relapse prevention treatment has a spillover effect on the quality of parent-child relationships. Moreover, we explore the carer’s attitudes about parent-child relationships and child gaming, as well as experiences of the treatment among the children, their carers, and the clinicians who carried out the treatment.

**Methods:**

This study is a 2-arm, parallel-group, early-stage randomized controlled trial with embedded qualitative components. Children aged 12-18 years who meet the criteria for problem gaming or internet gaming disorder will be randomized in a 1:1 ratio to either intervention (relapse prevention treatment) or control (treatment as usual), with a total of 160 (80 + 80) participants. The primary outcomes are measures of gaming and gambling behavior before and after intervention, and the secondary outcomes include child ratings of parent-child communication and family functioning. The study is supplemented with a qualitative component with semistructured interviews to capture participants’ and clinicians’ experiences of the relapse prevention, as well as attitudes about parent-child relationships and parenting needs in carers whose children completed the treatment.

**Results:**

The trial started in January 2022 and is expected to end in December 2023. The first results are expected in March 2023.

**Conclusions:**

This study will be the first randomized controlled trial evaluating relapse prevention as a treatment for child and adolescent problem gaming and internet gaming disorder in Sweden. Since problem behaviors in children interact with the family context, investigating parent-child relationships adjacent to the treatment of child problem gaming and internet gaming disorder is an important strength of the study. Further, different parties, ie, children, carers, and clinicians, will be directly or indirectly involved in the evaluation of the treatment, providing more knowledge of the treatment and its effect. Limitations include comorbidity in children with problem gaming and internet gaming disorder and challenges with the recruitment of participants.

**Trial Registration:**

ClinicalTrials.gov NCT05506384 (retrospectively registered); https://clinicaltrials.gov/ct2/show/NCT05506384

**International Registered Report Identifier (IRRID):**

DERR1-10.2196/44318

## Introduction

### Background

In 2013, internet gaming disorder, a syndrome of dysfunctional gaming behaviors that result in distress and affect personal, social, and educational functioning, was included in the appendix of the Diagnostic and Statistical Manual of Mental Disorders, Fifth Edition (DSM-5) [1] as a disorder that needed further research. Although the prevalence of internet gaming disorder is generally low, an increasing number of children who play digital games exhibit problematic gaming behaviors [2], which often result in dysfunctional social behaviors and mental health problems [3]. Delivering high-quality treatment for children with internet gaming disorder is key. This protocol describes a Swedish research project in which we will implement and evaluate an intervention labeled relapse prevention among children at Swedish child and youth psychiatry clinics.

Gaming, or playing offline or online digital games, has received a lot of attention from researchers and professionals in recent years. In Sweden, 68% of Swedish 13-16–year-old children and 55% of 17-18–year-old adolescents play computer games every day [4]. Gaming has become a common everyday arena where children and young people interact and socialize with others. Although some research suggests that gaming could be associated with more positive psychological outcomes, such as a stronger sense of belonging [5] and higher intelligence [6], other studies indicate that gaming can be linked to poor developmental outcomes such as physical and mental illness in adolescence [3]. Indeed, a small proportion of those who play digital games show a problematic development trajectory, similar to that of substance addiction, which is one of the reasons for including internet gaming disorder as an addiction diagnosis in the DSM-5 [1]. The diagnosis is phrased as “a persistent and recurrent use of the Internet to engage in games, often with other players, leading to clinically significant impairment or distress as indicated by five (or more) of the following criteria in a 12-month period: Preoccupation with gaming, withdrawal symptoms when gaming is taken away or not possible (sadness, anxiety, irritability), giving up other activities or continuing to game despite problems” [1]. Although the prevalence of problem gaming and internet gaming disorder in Swedish children and adolescents is to date unknown, a large European study with 12,938 children of 14-17 years of age reports that 5.1% exhibit problem gaming behaviors by fulfilling up to 4 criteria for internet gaming disorder, whereas 1.6% meet all the criteria for internet gaming disorder [7]. As many digital games are aimed at children, whose cognitive development means they are not always well equipped to deal with the instant gratification of most computer games [8], it is expected that problem gaming among children and adolescents will rise.

A development in the clinical management of the more well-established diagnosis of gambling addiction involving money [9,10] has brought attention to the possible association or co-occurrence of gambling, digital gambling, and problem gaming [11], not least given the development of in-game features such as loot boxes, ie, game-related purchases with a chance-based outcome. Moreover, earlier studies suggest that important correlates to child and adolescent problem gaming are male gender [12], neuropsychiatric disorder [13], difficulties with cognitive regulation [14], and substance use [15]. In addition, as parent-child relationships play an important role in child development [16], relationship features such as parent-child bonds and parent-child communication seem to be critical in terms of the development of problem gaming and internet gaming disorder [17]. Children with close parent-child bonds and open parent-child communication could more easily be offered and accept support from their parents, which subsequently could help them control their gaming behaviors. These factors need to be acknowledged both in routine practice as well as in the development of treatment for children and adolescents with problem gaming or internet gaming disorder.

To date, there is no gold standard treatment for children and adolescents with internet gaming disorder. Cognitive behavioral therapy (CBT) is, however, often identified as a first-line treatment for problem gaming, offering patients help with recognizing triggers and cues for gaming, and for understanding and controlling their gaming behaviors [18]. A CBT-based form of treatment, relapse prevention [19,20], has been developed as a treatment for child and adolescent problem gaming and internet gaming disorder. The relapse prevention program, which includes 3 to 8 sessions, has a motivational and relapse-prevention approach [19] where the therapist explores the target, ie, the problem behavior of the individual. Such program is traditionally used as a tertiary (or indicated) intervention strategy, meaning that such preventive effort is used to alleviate the impact of an ongoing problem and to prevent more complications. Relapse prevention treatment is often provided in outpatient clinics, for reducing the likelihood of relapsing into addiction and substance abuse. It is considered among the most effective preventive efforts for substance addiction [20] and works by teaching the individual to identify both internal and external cues to prevent future relapses in similar situations. We hypothesize that by targeting these mechanisms, the treatment could also be effective in treating children and adolescents with problem gaming and internet gaming disorder, because of the similarities of these behavioral addictions to substance addiction.

The effect of an intervention such as relapse prevention could, however, be dependent on the general quality of parent-child relationships and interactions between parents and their adolescent children, such that adolescents with strong family bonds and open parent-child communication would be likely to have more promising outcomes than adolescents with poor family bonds and communication. In that sense, the parent-child relationship could be an important moderator for the effectiveness of an intervention. In addition, given that adolescent development happens in interaction with their environment, such as family and more specifically parents [16], it is also possible that an intervention aimed at adolescents would also have a spillover effect on their environments where parent-child relationships are included. In that sense, not only would the individual benefit from such an effort, but the entire family would as well. Although such ideas seem compelling, they have not been tested in a context of gaming or relapse prevention.

### Aim and Objectives

The aim of the study is twofold: (1) to evaluate a relapse prevention treatment for patients with problem gaming and internet gaming disorder recruited from child and youth psychiatry clinics across southern Sweden (Region Skåne) and (2) to test whether the quality of parent-child relationships plays a role for the effect of relapse prevention treatment and vice versa—whether the relapse prevention treatment has a spillover effect on the quality of parent-child relationships.

The specific objectives are to:

1. Undertake an internal pilot to assess the recruitment and feasibility of delivering the treatment in a child and youth psychiatric clinic.
2. Conduct a randomized control trial (RCT) to determine the effectiveness of relapse prevention on child and adolescent problem gaming and internet gaming disorder.
3. Examine the moderating role of the parent-child relationship, including parent-child bonds and communication on the effect of relapse prevention on child and adolescent problem gaming and internet gaming disorder.
4. Investigate the effect of completed relapse prevention treatment on parent-child relationships
5. Introduce a qualitative component to address:
  The subjective experiences of relapse prevention treatment among children and adolescents who took part in the program and their view of problematic gaming
  Perceived attitudes about parent-child relationships and parenting needs in carers whose children accomplished the relapse prevention treatment
  The feasibility of the treatment among clinicians who delivered the program

## Methods

### Design

This study is a 2-arm, parallel-group, early-stage RCT with embedded qualitative components. The internal pilot will determine the recruitment and feasibility of the treatment. The recruitment start date is January 1, 2022, and the end date is December 30, 2022.

### Recruitment and Eligibility

The trial and the recruitment will be carried out in several outpatient child and youth psychiatric clinics in Region Skåne in southern Sweden. As part of routine practice, all children (ages 12-18 years) coming for their first visit to 4 child and youth psychiatric units in Region Skåne will be screened for gaming or gambling behaviors. Children will be eligible to be included in the trial if they are aged 12-18 years and referred to outpatient child and youth psychiatric clinics in Region Skåne, meet criteria for problem gaming and internet gaming disorder, and have capacity to provide written informed consent. Children below the age of 15 years need to have their caregivers’ consent. Children will be excluded if they do not speak Swedish. The clinicians who meet children during their first visit to the child and youth psychiatric unit will inform and invite the eligible children to participate in the study.

### Randomization

**Figure 1:**
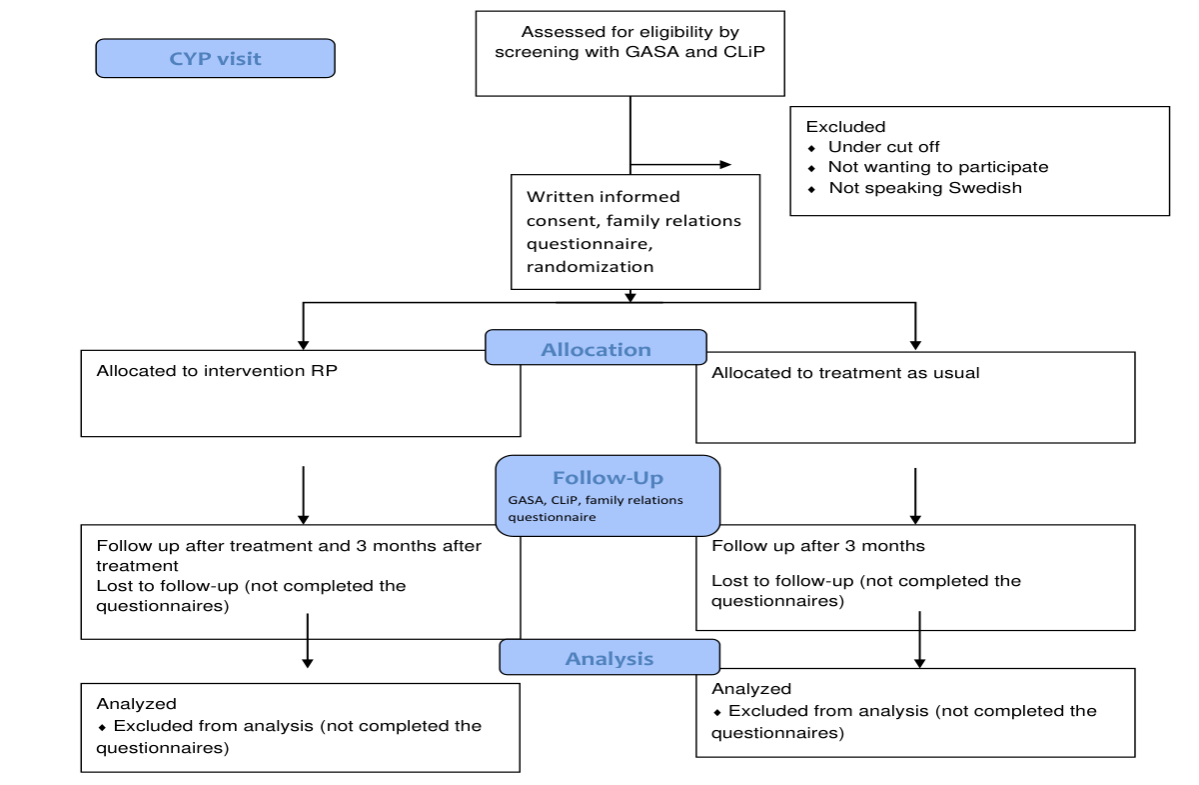
Flow diagram. CYP: Child and Youth Psychiatric Clinic; CLiP: Control, Lying, and Preoccupation; GASA; Game Addiction Scale for Adolescents; RP: relapse prevention.

Participants will be randomized in a 1:1 ratio to either intervention or control. The study will include a total of 160 participants, applying a random allocation sequence using the “chit method” by preparing 160 chits of paper indicating either control or treatment [21]. Each patient will be allocated to a condition (control or treatment), and the chit will not be replaced if the patient drops out of the study. The allocation to treatment and control arms may thus be uneven at certain times during the trial, but the end result of randomization will result in an equal distribution between control and treatment. It will not be possible to blind participants, clinicians, or supervising researchers to randomization allocation. The control group will receive “treatment as usual” at their home clinic. For an overview of recruitment and randomization, see the flow diagram in Figure 1.

### Intervention

There is at this time a lack of consensus regarding treatment methods for problem gaming [22], in part due to a lack of clinical research [23]. However, together with 4 experienced psychologists in the field, we developed a manual drawing on previous knowledge in the field of addiction and the field of child and adolescent psychiatric treatment. Relapse prevention for alcohol and substance abuse was used as a theoretical underpinning [20] to develop a manual with 2 aspects in mind: adapting the blurbs and examples for children and adolescents—in part by including a fictionalized adolescent when demonstrating the theme of the particular session; and by structuring the sessions so that they could be easily adapted to fit the participant’s primary problem behavior—gaming or gambling. Patients who meet the criteria for disordered gambling or gaming (according to tentative criteria from the DSM-5) will be offered the opportunity to participate in a relapse preventive treatment intervention at their local clinic or where applicable at an adjacent clinic. The treatment will be administered in an individual format and consist of 7 to 9 sessions of 45 minutes over a period of 7 to 9 weeks. The treatment will be offered to participants both in person and via video link to facilitate participation for children and adolescents living further away from the participating clinics.

The treatment consists of three parts: (1) setting goals, (2) understanding and identifying high-risk situations and problem behaviors, and (3) consolidating the new activity schedule and identifying future high-risk behaviors. The first part is focused on examining the patient’s undesirable behavior, his/her motivation for change, and establishing goals with the treatment (this part primarily draws on motivational interviewing). This part of the treatment therefore varies between 1 to 3 sessions as participants may have different levels of motivation for changing their primary problem behavior, which is why some participants may require 1 or 2 extra sessions at the start of the treatment. The second part (drawing more from traditional CBT techniques) consists of exploring problematic situations using functional analysis; identifying high-risk situations and events, emotions, and cognitions that induce the problematic gaming behavior or result in a relapse; and managing game time with activity scheduling and practicing problem-solving skills. The final part consists of recognizing early warning signals that may indicate that the primary problem behavior is more likely to occur and consolidating the parts of the treatment that have been the most helpful in maintaining the new activity schedule.

Clinicians (psychiatrists and psychologists) with training in CBT will beforehand be educated in relapse prevention and will throughout the treatment be supervised by a clinician with vast experience of treating adults with gambling and gaming addictions.

### Sample Size

Patients who have agreed to participate in the treatment study will be randomized in a 1: 1 ratio between intervention and control group. In our power calculation, we estimated that 40% in the intervention group and 20% in the control group will see improvement through the treatment offered in different groups, and after this treatment fall under the cutoff for problem gaming. With these figures, 160 (80 + 80) patients must be included in the treatment study for us to be able to demonstrate a significant difference with sufficient power. Given the large number of patients in pediatric and youth psychiatry in the region and the likely high incidence of problem behaviors in the population, we believe it is possible to reach this number of participants in the study.

### Ethics Approval

The study was reviewed and approved by the Swedish Ethical Review Authority (Ref 2019-04797, December 13, 2019). Subsequent amendments have been approved (Ref 2021-05592-01, January 3, 2021; Ref 2022-01289-02, March 15, 2022).

### Informed Consent

After eligibility is confirmed, written and verbal information about the study will be provided to all participants according to the Swedish Act concerning the Ethical Review of Research Involving Humans (SFS 2003:460). All patients, their carers, and clinicians who verbally agree to take part in the project will be provided with a consent form enabling them to provide written consent. For patients below the age of 15 years, a carer’s written consent will be needed for their children to take part in the study. All participants will be informed that their partaking in the study is voluntary; their data would be handled with strict confidentiality; results will be reported on a group level, which means that individual participants will not be identifiable; and they are free to withdraw from the study at any time without reporting a reason for withdrawal. Please see Multimedia Appendix 1 for details. The trial intervention is similar to other clinical practices offered in child and youth psychiatric clinics, which is why we consider the risks with the trial as minimal.

### Measures and Data Collection

Background information, including gender, age, housing situation, and diagnosis, as well as primary outcome measures assessing gaming and gambling problems, will be collected via the platform “Blå appen” (“Blue application” in Swedish). Blå appen is a digital platform developed by the child and youth outpatient department in Region Skåne, distributing and summarizing online self-rated questionnaires. It is used throughout Swedish child and youth psychiatry to facilitate the usage of self-rated questionnaires to patients. The secondary outcome measures, including parent-child communication and connectedness and family climate, will be administered in paper format after the child has provided consent to the study. Because the secondary outcomes measures assess general parent-child interactions, specific questions about gaming will also be added. The patients who consent to the RCT will rate their gaming and gambling activity as well as their parent-child relationships before and after the treatment. The “treatment at usual” arm will do the rating of gaming and gambling activity as well as their parent-child relationships at the inclusion and 3 months after the inclusion to the study. The following primary outcome measures will be used:

- The Game Addiction Scale for Adolescents [24] applies to gaming behavior during the previous 6 months based on 7 items. Each question covers one criterion in the DSM-5, answered on a 5-point Likert scale ranging from 1 (never) to 5 (very often) and should according to the developer be counted as endorsed when rated 3 or higher.
- The Diagnostic Screen for Gambling Problems–Control, Lying, and Preoccupation (NODS-CLiP) [25] is the shorter form of the NODS [26], which assesses gambling problems. NODS yield a score ranging from 0 to 10, corresponding to the DSM-4 criteria for gambling, where a score of 5 or more qualifies as pathological, a score of 3-4 corresponds to the subclinical syndrome of problem gambling, and scores of 1-2 corresponds to an “at-risk” status. NODS-CLiP comprises the 3 NODS items that best describe problem gambling, with 3 NODS questions pertaining to loss of Control, Lying, and Preoccupation—the “CLiP.”

The following secondary, somewhat adapted, outcome measures will be used:

- Parent-child communication [27] includes six subscales: (1) parent knowledge of child activities (eg, “Do your parents know how much time you spend on gaming?”); (2) parent control (eg, “How often do your parents set rules about your gaming activities?”); (3) parent solicitousness (eg, “How often do your parents ask you about your gaming activities?”); (4) child disclosure (eg, “Do you tell your parents about your gaming activities?”); (5) child secrecy (eg, “Do you hide a lot from your parents in terms of your gaming activities?”); and (6) child feelings of being overly controlled (eg, “Do you think your parents want to know too much about your gaming activities?”). Items are rated on a 5-point Likert scale ranging from 1 (often) to 5 (never).
- Family climate [28] includes 2 subscales, family cohesion and conflict. Although family cohesion assesses the bonds between family members (eg, “In my family, we help and support each other”), family conflict assesses the conflicts between family members (eg, “In my family we often fight with each other”). Items are rated on a 4-point scale, ranging from 1 (not true at all) to 4 (very true).

### Data Management

Personal and identifiable data will be collected from patients. Data will be kept confidential and managed in accordance with the Data Protection Act, General Data Protection Regulation policies and Swedish Act concerning the Ethical Review of Research Involving Humans (SFS 2003:460). Data will be held on services located within the Region Skåne databases, stored and secured both physically (in locked cabinets designed for the purpose) and electronically (behind firewalls), and be accessible to the study team only.

### Statistical Analysis

Preliminary analyses will be conducted with regression analyses, paired sample *t* tests and ANOVAs. Analyses of pre- to postintervention change will be based on probability modeling using both unadjusted and adjusted models with 95% CIs. Appropriate interaction terms will be included to test subgroup differences in the models [29]. Analyses will be performed in Mplus [30].

### Qualitative Component

In addition to the RCT, the study will be supplemented with a qualitative component with semistructured individual interviews in order to capture participants’ and clinicians’ experiences of the treatment, as well as attitudes about parent-child relationships and parenting needs in carers whose children completed the relapse prevention treatment. Specifically, the interviews with the children will focus on the children’s meaning-making of problem gaming, their experiences of the treatment, as well as their reflections on parent-child relationships before and after treatment. The interviews with the clinicians will focus on the clinicians’ experiences of carrying out the treatment, as well as their understanding of problem gaming. Finally, the interviews with the carers will focus on carers’ attitudes in terms of parent-child relationships before and after treatment of the child, as well as their reflections on potential parenting support needs in terms of their child’s gaming.

All interviews will be conducted by staff with knowledge of qualitative interview methodology. Each interview template will be pilot-tested prior to data collection. The respondents will be offered participation through a physical or digital interview. After the collection phase, the generated data will be analyzed with the help of thematic analysis based on the recommendations by Braun and Clarke [31].

### Dissemination

Findings from the study will be published in peer-reviewed journals and presented at local, national, and international conferences; workshops with key stakeholders; and interviews, pods, seminars, and lectures with general audiences. Results will also be reported to the funders.

The trial was retrospectively registered on ClinicalTrials.gov (NCT05506384; date of registration: April 13, 2022) due to administrative overload.

## Results

The trial started in January 2022 and is expected to end in December 2023. The first results are expected in March 2023.

## Discussion

### Expected Findings

In this research project, we will evaluate relapse prevention as a treatment for children and youth (aged 12-18 years) showing signs of problem gaming and internet gaming disorder. In addition, we will also test the role of parent-child relationships for the effects of treatment and include a qualitative component involving interviews with children, carers, and clinicians in order to gain a deeper understanding of the treatment and its anticipated effects.

CBT-based forms of treatment, such as relapse prevention [19,20], typically used as a treatment for substance addiction [20], has been adapted to the treatment of child and youth problem gaming and internet gaming disorder. The goal of the treatment is to help the individual to identify both internal and external cues to prevent future relapses in similar situations. More specifically, the relapse prevention treatment is thought to help children to recognize triggers and cues for gaming, as well as to understand and control their gaming behaviors. Therefore, we hypothesize that by targeting these mechanisms, the treatment could be effective in treating children and adolescents with problem gaming and internet gaming disorder. As children are a part of a family system [16], it is likely that aspects of parent-child relationship, such as communication and emotional bonds, would have impact on child gaming behaviors [17]. Acknowledging parent-child relationships as being an important factor for child behavioral development, we hypothesize that the quality of parent-child relationships will play role for the effect of the treatment in a sense that the treatment will be more efficient for children reporting stronger parent-child bonds and communication. In addition, as children and parents interact in a dynamic manner [16], we also expect that the treatment will have spillover effect on parent-child relationships. We also have exploratory components to the projects. As previous research on user acceptability and satisfaction with relapse prevention as a treatment for problem gaming and internet gaming disorder is lacking, we explore how children and their carers, as well as clinicians who carried out the treatment, experience relapse prevention as a treatment for problem gaming and internet gaming disorder among children and youth. In addition, we also explore carers’ attitudes in terms of parent-child relationships before and after treatment of the child, as well as their reflections on potential parenting support needs in terms of their child’s gaming.

### Limitations and Strengths

There are however some limitations to the study. The trial is not blinded, which may have an impact on both the patients’ behavior as well as clinicians’ practices. On the other hand, blinding does not necessarily ensure internal or external validity of the results [32], which is why we consider this limitation minor. As neuropsychiatric disorder is one of the major risk factors in problem gaming and internet gaming disorder [13], we expect that many of the patients included in the trial with be diagnosed with a neuropsychiatric disorder. This could be a potential limitation of the trial as the effect of the treatment may be affected by comorbidity in children with problem gaming or internet gaming disorder. Another possible limitation is the difficulty with recruiting patients as well as the potential dropout. Many children who game may not be aware of the problems that their gaming is imposing on their everyday lives, which is why they may not be likely to seek help or accept the given support. In addition, they may not be motivated to be engaged in the treatment. Therefore, we offer motivational sessions in the beginning of the treatment. Despite these limitations, there are several strengths to be noted. To our knowledge, this is the first RCT study testing the effect of relapse prevention on child and adolescent problem gaming and internet gaming disorder. As an increasing number of children engage in gaming [4,24], for some children, this activity may include significant problems [6,22] that would need attention from the clinics. Evaluating the efforts made by the clinics may provide more knowledge of the treatment of problem gaming in children. As problem behaviors in children interact with the family context, investigating parent-child relationships adjacent to the treatment of child problem gaming or internet gaming disorder is another important strength of the study. Further, this study includes a qualitative component involving different parties directly or indirectly involved in the treatment, ie, children, carers, and clinicians. Interviews with children, carers, and clinicians may help gaining knowledge of how the treatment is perceived by the individual who is directly involved in the treatment, the adults who take part in the child’s life on a daily basis, as well as the clinicians who are important stakeholders of the treatment.

### Conclusion

To conclude, in this project, we will evaluate relapse prevention as a treatment for children and youth (aged 12-18 years) showing signs of problem gaming and internet gaming disorder. The treatment will be evaluated in an RCT. The measures will include gaming frequency and gaming experiences, as well as perceived parent-child relationships and parent-child communication in order to understand the possible of role of parent-child relationship for the effect of the treatment. In addition, the study will also include a qualitative component involving interviews with children, carers, and clinicians in order to gain a deeper understanding of the treatment and its anticipated effects, as well as the parenting support needs the carers express. The results of the project will inform the development of practices in child and youth psychiatric clinics, putting focus on both children and their carers as important stakeholders in the practices.

## Appendix

Multimedia Appendix 1 Information letters and consent forms.

Multimedia Appendix 2 Peer review report by Swedish Research Council for Health, Working Life and Welfare / Forskningsrådet för hälsa, arbetsliv och välfärd - FORTE (Stockholm, Sweden).

## Abbreviations

CBT: cognitive behavioral therapy
DSM-5: Diagnostic and Statistical Manual of Mental Disorders, Fifth Edition
NODS-CLiP: The Diagnostic Screen for Gambling Problems–Control, Lying, and Preoccupation
RCT: randomized controlled trial

## Data Availability

The data sets generated and/or analyzed during the current study are not publicly available because this study’s ethical review does not allow for study data to be in a public repository. Requests to access the datasets should be addressed to ECK or SK.
