# Supplementary material for: Relapse Prevention Therapy for Problem Gaming or Internet Gaming Disorder in Swedish Child and Youth Psychiatric Clinics: Protocol for a Randomized Controlled Trial
Source: JMIR Res Protoc. 2023 Jan 5;12:e44318. doi: 10.2196/44318 (PMC9853338; doi:10.2196/44318)
Supplement: Multimedia Appendix 1 [file resprot_v12i1e44318_app1.pdf]

A treatment study for problematic gaming at Child- and Youth Psychiatry in Skåne

Supplementary material 1ab-6ab

1a – information about RCT study – for guardians
1b – consent for guardians
2a – information about RCT study – for children under the age of 15
3b – information about RCT study – for children above the age of 15
2b + 3b – consent for children
4a – information about interview study – for parents
4b – consent for parents
5a – information about interview study – for children above the age of 15
5b – consent for children
6a – information about interview study – for clinicians
6b – consent for clinicians

A treatment study for problematic gaming at Child- and Youth Psychiatry in Skåne

S1a

Information for Guardians

We would like to ask your child whether he/she wants to participate in a research project. In this document you will receive information about the project and what participation would involve. Your child will receive another information letter that is age appropriate.

More about the project and why we want your child to participate
Gaming has become a common activity for many children and young people but may pose problems for some children. The purpose of this project is to study a new treatment for gaming addiction in young people enrolled at Child- and Youth Psychiatry (BUP). The project owner is Child- and Youth Psychiatry in Region Skåne. The project owner is the organization that is responsible for the project. The project is approved by the ethics board with the reference number 2021-05592-02.

How will the project be done?
We will have two groups in the project; one treatment group and one group that will not get treatment. We call the last group the "control group". Your child will be randomly assigned to belong to either the new treatment group or the control group. At the start-up, both groups answer questions about their relationship with their parents/guardians and about their computer gaming. The questions take about 20 minutes to answer. The control group receives the usual treatment at Child- and Youth Psychiatry. After the treatment series, your child gets to answer the same questions about their relationship to their parents/guardians and about their computer gaming as they did on their first visit to BUP; time required 20 minutes. This applies regardless of whether they belong to the treatment group or the control group.

Possible consequences of and risks involved in participating in the project
We believe that the risks involved in participating in the project are low. It may be perceived that it is stressful to answer questions in a questionnaire. Therefore, we have tried to keep the forms short. The child can choose whether they want to skip a few questions. Participation in the study is completely voluntary and can be discontinued at any time a participant wishes, without giving any reason. The care your child receives at Child- and Youth Psychiatry will of course not be affected irrespective of whether the child is not allowed to participate, wants to participate in the study, or chooses to discontinue participation.

What happens to my data?
Only the responsible researchers who have access to the completed questionnaires, and no staff from Child- and Youth Psychiatry, will be able to see the survey responses. Only researchers in the project will be in charge of the material we collect. The coded responses from the surveys will be presented at group level. No information that can be linked to an individual is disclosed.

In the study, your child's personal data is replaced with a code number. The data is saved in order to make it possible for participants to discontinue their participation in the study, even after the questionnaires have been filled in. Region Skåne is responsible for the handling of personal data.

The children's responses and results will be processed so that unauthorized persons cannot access them. The person responsible for personal data for the study is Emma Claesdotter-Knutsson.

According to the EU General Data Protection Regulation, you have the right to access the information about your child handled in the study free of charge, and if necessary, have any errors corrected. You can also request that data about your child be deleted and that the processing of the child's personal data be restricted. If you want to access the information, please contact Emma Claesdotter-Knutsson; BUP Lund; Sofiav 2E; 22241 Lund; 0768871765. The Data Protection Officer can be reached via BUP Operations Manager Linda Wehlin 0768870066. If you are dissatisfied with how your child's personal data is processed, you have the right to lodge a complaint with the Swedish Privacy Authority, which is the supervisory authority.

How do I get information about the results of the project?
Results of the study will be published in scientific journals and also in daily media. Participants can also receive information about the results of the study by contacting the project manager in writing.

Insurance coverage
Region Skåne's insurance coverage applies throughout the study.

Participation is voluntary

Your child's participation is voluntary, and they can choose to cancel participation at any time. If they choose not to participate or want to cancel their participation, they do not need to state why, nor will it affect their future care or treatment.

If they wish to cancel their participation, you should contact the person responsible for the project (see below).

Responsible for the project
The study is conducted by Child- and Youth Psychiatry (BUP) in Lund and Addiction Center in Malmö; Region Skåne. If you have questions about the study, you are welcome to contact the study's responsible researchers.

Emma Claesdotter-Knutsson, med dr, Specialist in child and youth psychiatry, Consultant, BUP Lund, Sofiav 2E; 222 41 Lund,
Emma.claesdotter-knutsson@med.lu.se 0768871765

Anders Håkansson, professor, Lund University, Psychiatric Addiction Center Malmö,
anders_c_hakansson@med.lu.se

A treatment study for problematic gaming at Child- and Youth Psychiatry in Skåne

S1b

Guardian's consent for children's participation in the study "Treatment study for gaming addiction at Skåne Child- and Youth Psychiatry".

I have received oral and written information about the purpose of the research project and been given the opportunity to ask questions and have them answered. I get to keep the written information.

☐ I consent to my child participating in the study "Treatment study for gaming addiction at Skåne Child- and Youth Psychiatry"

Consent for:

(child's name): ____

(child's date of birth): ____

Please turn page!

Guardian 1

/Signature: ______________________________ Date: ____________________

Printed name : ______________________________

I have sole custody of my child: ☐ Yes ☐ No **If No, please also fill in guardian 2**

A treatment study for problematic gaming at Child- and Youth Psychiatry in Skåne

Guardian 2

Signature: ____ Date: ____

Printed name: ____

A treatment study for problematic gaming at Child- and Youth Psychiatry in Skåne

S2a

Information to participants

<u>For young people under the age of 15</u>

More about the project and why we want you to participate

Gaming has become a common activity for many children and young people. We would like to ask you if you would like to be part of a study that examines a new way to treat computer game addiction.

Here you will find information about the project and what it means to participate. We want to study a new treatment for gaming addiction in young people who are enrolled at Child- and Youth Psychiatry (BUP). The project owner is Child- and Youth Psychiatry in Region Skåne. The project owner is the organization that is responsible for the project. The project is approved by the ethics board with the reference number 2021-05592-02.

How will the project be done?

We will have two groups in the project; one treatment group and one group that will not get treatment. We call the last group the "control group". You will be randomly assigned to belong to either the new treatment group or the control group.

All of you will answer questions about your relationship to your parents/guardians and about your computer gaming. The questions take about 20 minutes to answer. The control group receives the usual treatment at Child- and Youth Psychiatry. After the treatment series, you will get to answer the same questions about your relationship to your parents/guardians and about your computer gaming as you did on your first visit to BUP; time required 20 minutes. This applies regardless of whether you belong to the treatment group or the control group.

Possible consequences of and risks involved in participating in the project

We believe that the risks involved in participating in the project are few. It may be perceived that it is stressful to answer questions in a questionnaire. Therefore, we have tried to keep the forms short. You can choose whether you want to skip questions if you find them difficult.

What happens to my data?

Only the responsible researchers will be able to read the survey responses. In the study, your personal data is replaced with a code number.

How do I get information about the results of the project?

Results of the study will be published in scientific journals and also in daily media. You, as a participant, can also receive information about the results of the study by contacting the project manager in writing.

Insurance coverage

Region Skåne's insurance coverage applies throughout the study.

A treatment study for problematic gaming at Child- and Youth Psychiatry in Skåne

Participation is voluntary
You decide whether you want to participate. You can change your mind at any time and drop out without saying why. The care you receive at BUP will not be affected if you drop out. Since you are under the age of 15, you must have the consent of your guardians to participate in the study. Even if they say yes, it is still you who decides whether you want to participate or not.

Responsible for the project
If you have questions about the study, you are welcome to call or write:

Emma Claesdotter-Knutsson, med dr, Specialist in child and youth psychiatry, Consultant, BUP Lund, Sofiav 2E; 222 41 Lund,
Emma.claesdotter-knutsson@med.lu.se 0768871765

Anders Håkansson, professor, Lund University, Psychiatric Addiction Center Malmö, anders_c_hakansson@med.lu.se

A treatment study for problematic gaming at Child- and Youth Psychiatry in Skåne

S2b + S3b

Children and young people under the age of 17

Consent for participation in the study “Treatment study for gaming addiction at Skåne Child- and Youth Psychiatry”.

I have received oral and written information about the purpose of the research project and been given the opportunity to ask questions and have them answered. I get to keep the written information.

☐ I consent my participation in the study “Treatment study for gaming addiction at Skåne Child- and Youth Psychiatry”

Consent for:

(Your name): ____

(Your date of birth): ____

Signature: ____ Date: ____

Printed name: ____

A treatment study for problematic gaming at Child- and Youth Psychiatry in Skåne

S3a

Information for participants

For young people between the ages of 15 and 17

We would like to ask you if you would like to participate in a research project. This document provides information about the project and what it means to participate.

More about the project and why we want you to participate
Computer gaming is an increasingly common activity for many children and adolescents. While some play only occasionally, others play very often. The purpose of this project is to investigate a new treatment method for computer game addiction in young people enrolled at Child- and Adolescent Psychiatry (BUP). We would like to ask you if you would like to be part of a study that examines a new way to treat computer game addiction.

The project owner is Child- and Youth Psychiatry in Region Skåne. The project owner is the organization that is responsible for the project. The project is approved by the ethics board with the reference number 2021-05592-02.

How will the project be done?
We will have two groups in the project, one treatment group and one group that will not get treatment. We call the last group the "control group". You will be randomly assigned to belong to either the new treatment group or the control group.

At the start-up, both groups answer questions about their relationship with their parents and about their computer gaming. The questions take about 20 minutes to answer. The treatment group receives a series of conversations with a therapist. Each conversation is estimated to take 45 minutes. The control group receives the usual treatment at BUP. After the conversation series, you get to answer the same questions about your relationship with your parents and about your gaming as you did during your first visit to BUP; time required 20 min. This applies regardless of whether you belong to the treatment group or the control group.

Possible consequences of and risks involved in participating in the project
We believe that the risks involved in participating in the project are low. It may be perceived that it is stressful to answer questions in a questionnaire. Therefore, we have tried to keep the forms short. You can choose whether you want to skip a few questions. Participation in the study is completely voluntary and can be discontinued at any time a participant wishes, without giving any reason. The care you receive at Child- and Youth Psychiatry will of course not be affected if you choose not to participate in the study or should you choose to discontinue participation.

What happens to my data?
Only the responsible researchers who have access to the completed questionnaires, and no staff from Child- and Youth Psychiatry, will be able to see the survey responses. Your participation in the study does not affect your treatment at BUP. Only researchers in the project will be in charge of the material we collect. The coded responses from the surveys will be presented at group level. No information is disclosed that can be linked to an individual.

In the study, your personal data is replaced with a code number. The data is saved so that it will be possible for you as a participant to interrupt your participation in the study, even after the surveys have been filled in. Region Skåne is responsible for the handling of your personal data.

Your answers and your results will be processed so that unauthorized persons cannot access them. Emma Claesdotter-Knutsson is responsible for your personal data and is also responsible for the study. According to the EU General Data Protection Regulation, you have the right to access the information about you handled in the study free of charge, and if necessary, have any errors corrected. You can also request that data about you be deleted and that the processing of your personal data be restricted.

If you want to access the information, you can contact Emma Claesdotter-Knutsson; BUP Lund; Sofiav 2E; 22241 Lund; 0768871765. The Data Protection Officer can be reached via BUP Operations Manager Linda Wehlin 0768870066. If you are dissatisfied with how your personal data is processed, you have the right to lodge a complaint with the Swedish Privacy Authority, which is the supervisory authority.

How do I get information about the results of the project?

Results of the study will be published in scientific journals and also in daily media. Participants can also receive information about the results of the study by contacting the project manager in writing.

Insurance coverage

Region Skåne's insurance coverage applies throughout the study.

Participation is voluntary

Your participation is voluntary, and you can choose to cancel participation at any time. If you choose not to participate or want to cancel your participation, you do not need to state why, nor will it affect your future care or treatment.

If you wish to cancel your participation, you should contact the person responsible for the project (see below).

Responsible for the project

The study is conducted by Child- and Youth Psychiatry in Lund and Addiction Center in Malmö; Region Skåne. If you have questions about the study, you are welcome to contact the study's responsible researchers.

Emma Claesdotter-Knutsson, med dr, Specialist in child and youth psychiatry, Consultant, BUP Lund, Sofiav 2E; 222 41 Lund,
Emma.claesdotter-knutsson@med.lu.se 0768871765

Anders Håkansson, professor, Lund University, Psychiatric Addiction Center Malmö,
anders_c_hakansson@med.lu.se

A treatment study for problematic gaming at Child- and Youth Psychiatry in Skåne

S4a

Information for participants

<u>For parents/guardians</u>

We want to ask you if you would like to participate in an interview about how you feel the relationship between you and your child has been affected by your child undergoing treatment for problematic gaming, and what support you as a parent feel you need in relation to your child's gaming. In this document, you will receive information about the interview and about what it means to participate.

More about the project and why we want you to participate

The aim of the project is to evaluate a new treatment method for problematic gaming in young people, developed by Child- and Youth Psychiatry (BUP). As part of the evaluation, we also want to gain knowledge about what support the parent/guardian feels they need based on the child's gaming, and how the parent/guardian feels that the relationship with the child is affected by the child undergoing treatment. We would therefore like to ask you if you would like to participate in an interview about these questions.

The project owner is Child- and Youth Psychiatry in Region Skåne. The project owner is the organization that is responsible for the project. The project is approved by the ethics board with the reference number 2021-05592-02.

How will the interview be done?

We will interview the parent/guardian of the child who completed the treatment. The interviews are conducted by our research group and take about 45 minutes to complete. The questions concern your perceived need for support based on your child's gaming and how the relationship with your child looked before and after the child's treatment. We will ask to record the interviews to facilitate the interpretation and processing of the answers.

Possible consequences of and risks involved in participating in the project

We believe that the risks involved in participating in the project are low. The interview gives you the opportunity to describe what need for support you have experienced and how the child's treatment has affected your relationship, and thereby contributes to the development of better care for others. Participation in the interview is entirely voluntary and can be cancelled at any time you wish.

What happens to my data?

Only the responsible researchers have access to the interview material. The processed responses from the interviews will be presented at group level and no sensitive data will be reported individually. No information is disclosed that can be linked to an individual.

Your answers will be processed so that unauthorized persons cannot access them. Emma Claesdotter-Knutsson is responsible for your personal data and is also responsible for the study. According to the EU General Data Protection Regulation, you have the right to access the information about you handled in the study free of charge, and if necessary, have any errors corrected.

You can also request that data about you to be deleted and that the processing of your personal data be restricted. If you want to access the information, you should contact Emma Claesdotter-Knutsson; BUP Lund; Sofiav 2E; 22241 Lund; 0768871765. The Data Protection Officer can be reached via BUP Operations Manager Linda Wehlin 0768870066.

If you are dissatisfied with how your personal data is processed, you have the right to lodge a complaint with the Swedish Privacy Authority, which is the supervisory authority.

How do I get information about the results of the project?
Results of the study will be published in scientific journals and also in daily media. Participants can also receive information about the results of the study by contacting the project manager in writing.

Insurance coverage
Region Skåne's insurance coverage applies throughout the study.

Participation is voluntary

Your participation is voluntary, and you can choose to cancel participation at any time. If you choose not to participate or want to cancel your participation, you do not need to state why, nor will it affect the future care or treatment of your child.

If you wish to cancel your participation, you should contact the person responsible for the project (see below).

Responsible for the project
The study is conducted by Child- and Youth Psychiatry in Lund and Addiction Center in Malmö; Region Skåne. If you have questions about the study, you are welcome to contact the study's responsible researchers.

Emma Claesdotter-Knutsson, med dr, Specialist in child and youth psychiatry, Consultant, BUP Lund, Sofiav 2E; 222 41 Lund
Emma.claesdotter-knutsson@med.lu.se 0768871765

Anders Håkansson, professor, Lund University, Psychiatric Addiction Center Malmö, anders_c_hakansson@med.lu.se

S4b

For parent/guardian
Consent for participation in the interview within the framework of the study on problematic computer gaming with and without money at Child- and Adolescent Psychiatry, Skåne.

I have received oral and written information about the research project and its purpose and been given the opportunity to ask questions and to have them answered. I get to keep the written information.

I agree to participate in the interview ( )

Consent for:

(Your name):

(Your birth date):

Signature: ____ Date:______________

Printed name: ____

A treatment study for problematic gaming at Child- and Youth Psychiatry in Skåne

S5a

Information for participants

For young people between ages 15 and 18

We would like to ask you if you would like to participate in an interview about your experience of your treatment for problematic gaming. In this document, you will receive information about the interview and about what it means to participate.

More about the project and why we want you to participate

The purpose of the interview is to evaluate a new treatment method for gaming addiction in young people, developed by Child- and Youth Psychiatry (BUP). We also want to gain a better understanding of how young people who have undergone the treatment understand problematic gaming and how they experienced the treatment. We would therefore like to ask you if you would like to participate in an interview and tell us about your experience of the treatment.

The project owner is Child- and Youth Psychiatry in Region Skåne. The project owner is the organization that is responsible for the project. The project is approved by the ethics board with the reference number 2021-05592-02.

How will the interview be done?

All young people over the age of 15 who have undergone treatment for problematic gaming will be asked to participate in the interview. The interviews are conducted by our research team and take about 45 minutes to complete. The questions concern the themes "Experience of problematic gaming" and "Undergoing treatment for problematic gaming". We will ask to record the interviews to facilitate the analysis and processing of the answers.

Possible consequences of and risks involved in participating in the project

We believe that the risks of participating in the project are low. There is a possibility to perceive that it is stressful to answer questions about one's own attitudes and thoughts. Therefore, we will try to keep the interview as short as possible. You can also choose for yourself whether you want to skip a few questions. One advantage, however, is that the interview gives you the opportunity to give your opinion on the treatment and thereby contribute to the development of better care for others.

Participation in the interview is voluntary and can be interrupted at any time a participant wishes, without giving any reason. The care you receive at BUP will of course not be affected if you choose not to participate in the interview, or should you choose to cancel your participation.

What happens to my data?

Only the responsible researchers have access to the interview material. The processed responses from the interviews will be presented at group level and no sensitive data will be reported individually. No information is disclosed that can be linked to you as an individual.

Region Skåne is responsible for the handling of your personal data.

Your answers will be processed so that unauthorized persons cannot access them. Emma Claesdotter-Knutsson responsible for your personal data. According to the EU General Data Protection Regulation, you have the right to access the information about you handled in the study free of charge, and if necessary, have any errors corrected. You can also request that data about you be deleted and that the processing of your personal data be restricted. If you want to access the information, you can contact Emma Claesdotter-Knutsson; BUP Lund; Sofiav 2E; 22241 Lund; 0768871765. The Data Protection Officer can be reached via BUP Operations Manager Linda Wehlin 0768870066.

If you are dissatisfied with how your personal data is processed, you have the right to submit a complaint to the Privacy Authority, which is the supervisory authority.

How do I get information about the results of the project?
Results of the study will be published in scientific journals and also in daily media. Participants can also receive information about the results of the study by contacting the project manager in writing.

Insurance coverage
Region Skåne's insurance coverage applies throughout the study.

Participation is voluntary

Your participation is voluntary, and you can choose to cancel participation at any time. If you choose not to participate or want to cancel your participation, you do not need to state why, nor will it affect your future care or treatment.

If you wish to cancel your participation, you should contact the person responsible for the project (see below).

Responsible for the project
The study is conducted by Child- and Youth Psychiatry in Lund and Addiction Center in Malmö; Region Skåne. If you have questions about the study, you are welcome to contact the study's responsible researchers.

Emma Claesdotter-Knutsson, med dr, Specialist in child and youth psychiatry, Consultant, BUP Lund, Sofiav 2E; 222 41 Lund
Emma.claesdotter-knutsson@med.lu.se 0768871765

Anders Håkansson, professor, Lund University, Psychiatric Addiction Center Malmö, anders_c_hakansson@med.lu.se

A treatment study for problematic gaming at Child- and Youth Psychiatry in Skåne

S5b

For young people above the age of 15:
Consent for participation in an interview within the project on problematic computer gaming with and without money at Child- and Youth Psychiatry, Skåne.

I have received oral and written information about the research project and its purpose and been given the opportunity to ask questions and to have them answered. I may retain the written information.

● I agree to participate in the interview ( )

Consent for:

(Your name):

(Your date of birth)

Signature: ____ Date:_____________

Printed name: ____

S6a

Information for participants

For clinicians

We would like to ask you if you would like to participate in an interview about your experience of treatment for problematic gaming. In this document, you will receive information about the interview and about what it means to participate.

More about the project and why we want you to participate

The purpose of the interview is to evaluate a new treatment method for gaming addiction in young people, developed by Child- and Youth Psychiatry (BUP). We also want to gain a better understanding of how clinicians who have delivered the treatment understand problematic gaming and what their experiences are of the treatment. We would therefore like to ask you if you would like to participate in an interview and tell us about your experience of the treatment.

The project owner is Child- and Youth Psychiatry in Region Skåne. The project owner is the organization that is responsible for the project. The project is approved by the ethics board with the reference number 2021-05592-02.

How will the interview be done?

We will interview clinicians who have delivered Relapse Prevention as treatment for problem gaming or Internet gaming disorder. The interviews are conducted by our research team and take about 60 minutes to complete. The questions concern the themes "Understanding problem gaming" and "Delivering treatment for problematic gaming". We will ask to record the interviews to facilitate the analysis and processing of the answers.

Possible consequences of and risks involved in participating in the project

We believe that the risks of participating in the project are low. One advantage, however, is that the interview gives you the opportunity to give your opinion on the treatment and thereby contribute to the development of better care for others.

Participation in the interview is voluntary and can be interrupted at any time a participant wishes, without giving any reason.

What happens to my data?

Only the responsible researchers have access to the interview material. The processed responses from the interviews will be presented at group level and no sensitive data will be reported individually. No information is disclosed that can be linked to you as an individual.

Region Skåne is responsible for the handling of your personal data.

A treatment study for problematic gaming at Child- and Youth Psychiatry in Skåne

Your answers will be processed so that unauthorized persons cannot access them. Emma Claesdotter-Knutsson responsible for your personal data. According to the EU General Data Protection Regulation, you have the right to access the information about you handled in the study free of charge, and if necessary, have any errors corrected. You can also request that data about you be deleted and that the processing of your personal data be restricted. If you want to access the information, you can contact Emma Claesdotter-Knutsson; BUP Lund; Sofiav 2E; 22241 Lund; 0768871765. The Data Protection Officer can be reached via BUP Operations Manager Linda Wehlin 0768870066.

If you are dissatisfied with how your personal data is processed, you have the right to submit a complaint to the Privacy Authority, which is the supervisory authority.

How do I get information about the results of the project?
Results of the study will be published in scientific journals and also in daily media. Participants can also receive information about the results of the study by contacting the project manager in writing.

Insurance coverage
Region Skåne's insurance coverage applies throughout the study.

Participation is voluntary

Your participation is voluntary, and you can choose to cancel participation at any time. If you choose not to participate or want to cancel your participation, you do not need to state why, nor will it affect your future care or treatment.

If you wish to cancel your participation, you should contact the person responsible for the project (see below).

Responsible for the project
The study is conducted by Child- and Youth Psychiatry in Lund and Addiction Center in Malmö; Region Skåne. If you have questions about the study, you are welcome to contact the study's responsible researchers.

Emma Claesdotter-Knutsson, med dr, Specialist in child and youth psychiatry, Consultant, BUP Lund, Sofiav 2E; 222 41 Lund
Emma.claesdotter-knutsson@med.lu.se 0768871765

Anders Håkansson, professor, Lund University, Psychiatric Addiction Center Malmö, anders_c_hakansson@med.lu.se

A treatment study for problematic gaming at Child- and Youth Psychiatry in Skåne

S6b

For clinicians

Consent for participation in the interview within the framework of the study on problematic computer gaming with and without money at Child- and Youth Psychiatry, Skåne.

I have received oral and written information about the research project and its purpose and been given the opportunity to ask questions and to have them answered. I get to keep the written information.

- I consent to participate in the interview ( )

Consent for:

(Your name):

(Your date of birth):

Signature: ____ Date:_____________

Printed name: ____
