# Supplementary material for: Relapse Prevention Therapy for Problem Gaming or Internet Gaming Disorder in Swedish Child and Youth Psychiatric Clinics: Protocol for a Randomized Controlled Trial
Source: JMIR Res Protoc. 2023 Jan 5;12:e44318. doi: 10.2196/44318 (PMC9853338; doi:10.2196/44318)
Supplement: Multimedia Appendix 2 [file resprot_v12i1e44318_app2.pdf]

Following decisions are included in the file:

1. Craaford Foundation (Ref 20200862)
   please see p. 2 in this document. Point 4 is stating that Emma Clasdotter Knutsson receives funding)
2. Fanny Ekdahls Foundation (-)
   Please see p. 3-6 in this document. This is a contract between the foundation and Child and Youth Psychiatric Clinic – particularly point 1 and 2 are of interest stating that Emma Claesdotter Knutsson receives funding.
3. Svenska spels forskningsråd [Sweden's gaming company research council] (Ref 2020-003)
   please *see p. 7 in this document – the funding is received by Emma Claesdotter Knutsson*
4. Swedish Research Council for Health, Working Life and Welfare FORTE (Ref 2021-01696)
   please *see p. 8 in this document – the funding is received by Sabina Kapetanovic*

PROTOKOLL FÖRT VID STYRELSEMÖTE I FANNY EKDAHLS STIFTELSE FÖR PEDIATRISK FORSKNING 2019-12-11

Närvarande styrelseledamöter: Fanny Ekdahl, Nils Petter Ekdahl (ordförande), Monica Rosenqvist, Rolf Ljung

1. Nils Petter Ekdahl förklarade mötet öppnat.

2. Monica Rosenqvist redogjorde för förvaltningen av stiftelsens kapital, se sammanfattning bilaga 1.

3. Rolf Ljung redogjorde för de ansökningar om anslag ur stiftelsen som inkommit under årets ansökningstid.

4. Beslutades därefter att bevilja anslag åt Emma Claesdotter Knutsson och Ioannis Orfanos med vardera 200 000 kr. Uppdrogs åt Monica Rosenqvist att meddela de sökande om beslutet enligt samma modell som tidigare år samt åt Rolf Ljung att ta fram ett diplom som kan överlämnas till Emma Claesdotter Knutsson och Ioannis Orfanos.

5. Mötet avslutades och det uppdrogs åt Nils Petter Ekdahl att sammankalla ett nytt styrelsemöte före utgången av april månad 2020, då räkenskaperna för år 2019 ska fastställas.

Nils Petter Ekdahl

SVENSKA SPELS FORSKNINGSRÅD
för forskning om spel och förebyggande arbete mot spelproblem

AVTALSVILLKOR FÖR ERHÅLLANDE AV ANSLAG

1. Parter

1.1. Detta avtal har ingåtts mellan:

AB Svenska Spel, 621 80 Visby, org. nr: 556460-1812 (”Svenska Spel”)

Kontaktperson: Karin Granath.

BUP Lund, IKVL, Barnpsykiatri, Baravägen 1, 221 85 Lund, Org nr: 202100-3211(”Bidragsmottagaren”)

Kontaktperson: Emma Claesdotter-Knutsson

Ovan nämnda parter benämnda var och en för sig ”Part” och tillsammans ”Parterna” i detta avtal.

2. Bakgrund

2.1. Svenska Spels Forskningsråd (”Forskningsrådet”) för forskning om spel och förebyggande arbete mot spelproblem har utlyst utdelning av anslag till spelforskning.

2.2. Forskningsrådet har beslutat att bevilja Bidragsmottagaren bidrag för forskningsprojektet *”Spel och spelproblem med och utan pengar i en barn- och ungdomspopulation”(”*Forskningsprojektet”), översiktligt beskriven i **Bilaga 1 - Beskrivning av Forskningsprojektet**. Bidragsmottagaren erhåller totalt 2 143 190 kronor (fördelat i enlighet med **Bilaga 2 - Betalningsplan**) för Forskningsprojektet (”Forskningsanslaget”).

3. Bidragsmottagarens ansvar och åtaganden

3.1. Forskningsanslaget får disponeras i högst 3 år från första utbetalning. Forskningsrådet kan fatta beslut om att förlänga denna tidsperiod pga. exempelvis sjukdom, föräldraledighet eller annan enligt Forskningsrådet giltig orsak som föranleder avbrott i Forskningsprojektet.

3.2. Bidragsmottagaren ansvarar för att Forskningsanslaget används för Forskningsprojektet i enlighet med till ansökan bifogad projektplan. Ändringar avseende användningen av Forskningsanslaget och/eller ändringar i den tidsplan som anges i projektplanen kan medges efter skriftligt godkännande från Forskningsrådet

3.3. Bidragsmottagaren förvaltar Forskningsanslaget och är arbetsgivare för den eller de forskare som forskar eller deltar i Forskningsprojektet. Bidragsmottagaren ansvarar såsom arbetsgivare för inbetalning av arbetsgivaravgifter, sociala avgifter eller annan skatt eller avgift i samband med Forskningsprojektet.

3.4. Bidragsmottagaren ansvarar även för att Bidragsmottagaren innehar giltig F-skattsedel och det åligger Bidragsmottagaren att under hela avtalstiden vidmakthålla F-skattsedel. Om

genom myndighets beslut Svenska Spel skulle komma att anses vara arbetsgivare, eller för det fall att Bidragsmottagaren saknar giltig F-skattsedel, och om Svenska Spel blir ansvarig för att erlägga arbetsgivaravgifter, sociala avgifter, eller någon annan skatt, avgift eller pålaga, skall Bidragsmottagaren ersätta Svenska Spel härför med motsvarande belopp.

3.5. Bidragsmottagarens kostnader för administration och förvaltning av Forskningsanslaget får inte överstiga den andel av det ansökta forskningsanslaget som anges i ansökan.

3.6. Bidragsmottagaren är skyldig att utan dröjsmål meddela Forskningsrådet om annan finansiering av Forskningsprojektet finns eller tillkommer. För det fall annan finansiering förekommer och totalt anslag för Forskningsprojektet överstiger av Forskningsprojektet sökt anslag har Forskningsrådet rätt att minska Forskningsanslaget motsvarande den del som överstiger det sökta anslaget. Finansiering från parter som konkurrerar med Svenska Spel ska alltid godkännas skriftligen av Forskningsrådet.

3.7. Om Forskningsprojektet inte bedrivs eller om Forskningsanslaget inte används i överensstämmelse med vad som anges i ansökan och enligt detta avtal, har Forskningsrådet rätt att kräva återbetalning av Forskningsanslaget.

4. Rapportering och ekonomisk redovisning

4.1. Bidragsmottagaren skall inge till Forskningsrådet en slutlig ekonomisk redovisning av användningen av Forskningsanslaget senast 2023-12-31. Medel som inte har använts för sitt ändamål ska senast trettio (30) dagar efter slutredovisningen återbetalas till Svenska Spel. Vid försenad återbetalning äger Svenska Spel rätt att debitera dröjsmålsränta i enlighet med räntelagen (1975:635).

4.2. Bidragsmottagaren ska samtidigt med den slutliga ekonomiska redovisningen inge en redogörelse för Forskningsprojektets arbete och uppnådda resultat.

4.3. Om Forskningsprojektet sträcker sig längre än ett (1) år, skall rapportering och ekonomisk redovisning ske årligen och inges senast 31 december varje år, med slutredovisning senast den 2023-12-31 om inte annat överenskommes. Forskningsrådet har även rätt att begära in rapportering vid andra tidpunkter och Bidragsmottagaren skall inkomma med sådan rapportering inom skälig tid efter Forskningsrådets begäran.

4.4. Bidragsmottagaren och de forskare som utsetts att bedriva Forskningsprojektet skall i skälig omfattning stå till förfogande för att informera Forskningsrådet om sin verksamhet och sina resultat fram tills disputation och sex (6) månader därefter.

4.5. Bidragsmottagaren och Forskningsrådet skall gemensamt besluta om hur och när det slutliga resultatet av Forskningsprojektet (”Resultatet”) skall offentliggöras. Forskningsrådet har rätt att delta vid offentliggörandet av Resultatet. Oaktat föregående mening bestämmer Bidragsmottagaren själv när Resultatet ska publiceras i vetenskapliga tidskrifter förutsatt att Forskningsrådet i god tid informeras om tidsskift och datum för publicering.

5. Forskningsrådets åtaganden

Forskningsrådet skall vara Bidragsmottagaren behjälplig i frågor kring Forskningsanslaget.

6. Utbetalning av Forskningsanslaget

6.1. Beviljat Forskningsanslag utgör det totala belopp som Svenska Spel tillför Forskningsprojektet och inkluderar mervärdesskatt.

6.2. Forskningsanslaget ska utbetalas till Bidragsmottagaren i enlighet med **Bilaga 2 – Betalningsplan** eller annars i enlighet med vad som överenskommits mellan parterna, dock senast innan årsskiftet det år Forskningsanslaget beviljats. Forskningsrådet äger dock rätt att besluta om Forskningsanslaget skall utbetalas vid ett tillfälle eller såsom delutbetalningar.

6.3. Forskningsrådet har rätt att stoppa fortsatt utbetalning av Forskningsanslaget för det fall rapportering och/eller ekonomisk redovisning enligt punkt 4 ovan inte inkommit i tid eller underkänts av Forskningsrådet. För det fall rapportering och/eller ekonomisk redovisning underkänns, skall Bidragsmottagaren, så snart detta är möjligt, erhålla information om detta inklusive en skriftlig motivering med anledningen till underkännandet samt vad som behöver kompletteras för att godkännande skall erhållas.

6.4. Vid försenad betalning äger Bidragsmottagaren rätt att debitera dröjsmålsränta i enlighet med räntelagen, (1975:635). Försenad betalning på grund av förhållanden enligt punkten 6.3 ovan ger dock inte Bidragsmottagaren rätt att debitera dröjsmålsränta. Bidragsmottagaren äger inte rätt att debitera faktureringsavgift eller andra icke avtalade tilläggsavgifter.

7. Ägande- och nyttjanderätt

7.1. Rättigheterna till Resultatet ägs av Bidragsmottagaren/berörd forskare. Bidragsmottagaren skall dock låta Forskningsrådet ta del av och upplåter rätt till Forskningsrådet att fritt och för obegränsad tid nyttja Resultatet, inklusive de årliga rapporteringarna om Forskningsprojektet (samt överlåta sådan nyttjanderätt till tredjeman). Oaktat ovanstående accepterar Forskningsrådet att upphovsrätten till specifika publikationer och rapporter, framtagna utifrån Resultatet, kan komma att överlåtas till vetenskapliga tidskrifter i samband med publikation av sådana publikationer och rapporter. Forskningsrådet och Bidragsmottagaren skall överenskomma särskilt om rättigheterna och nyttjande till sådant material som Bidragsmottagaren/forskaren erhållit från Svenska Spel/Forskningsrådet för Forskningsprojektet.

7.2. Bidragsmottagaren skall i artiklar, vid presentationer av Forskningsprojektet och annan publicering om Forskningsprojektet och Resultatet ange att Forskningsprojektet genomförts med anslag från Forskningsrådet. Uttalanden gällande Forskningsrådets verksamhet i övrigt får ej göras utan Forskningsrådets på förhand skriftliga godkännande.

7.3. Bidragsmottagaren äger inte rätt att utnyttja Svenska Spels eller Forskningsrådets logotyp, varumärken eller kännetecken i sin verksamhet eller för annat ändamål än som Forskningsrådet uttryckligen har medgivit på förhand. Föregående mening gäller inte i de fall som anges i punkten 7.2 ovan såtillvida avser användandet av ”Svenska Spel” och ”Svenska Spels Forskningsråd”.

8. Personuppgifter

8.1. Bidragsmottagaren är införstådd med att Forskningsrådet kan komma att registrera information om sådana kontaktpersoner, företrädare för Bidragsmottagaren och/eller andra person som utpekas eller presenteras av Bidragsmottagaren i samband med detta avtal. Svenska Spels/Forskningsrådets behandling av personuppgifter i enlighet med ovanstående sker i enligt vid var tid gällande dataskyddslagstiftning och i syfte att fullgöra avtalet mellan Svenska Spel/Forskningsrådet och Bidragsmottagaren. Det åligger Bidragsmottagaren att i vederbörlig ordning informera sådana personer som berörs härav.

8.2. Bidragsmottagaren accepterar att följa vid var tid tillämplig dataskyddslagstiftning.

9. Force Majeure

9.1. Händelser utanför parts kontroll (s.k. Force Majeure) som förhindrar parts fullgörande av sina åtaganden enligt detta avtal, skall utgöra grund för befrielse från ansvar, dröjsmål, skadestånd och andra påföljder.

9.2. Part, som till befrielse från förpliktelse vill åberopa sådan omständighet som anges ovan, skall omgående skriftligen underrätta den andra Parten, med angivande av vilken omständighet som åberopas och den tidpunkt då hindret kan antas ha övervunnits.

10. Överlåtelse

Bidragsmottagaren får ej överlåta dennes rättigheter och skyldigheter i förhållande till Forskningsrådet enligt detta avtal till annat universitet, institution eller annan tredje part, utan Forskningsrådets på förhand skriftliga medgivande.

11. Ändringar och tillägg

Alla ändringar och tillägg till detta Avtal ska vara skriftliga och undertecknade av båda Parter för att vara giltiga.

12. Tvistelösning, tillämplig lag

12.1. Svensk lag skall äga tillämpning på detta avtal.

12.2. Tvist i anledning av detta avtal ska slutligt avgöras i allmän domstol med Stockholms tingsrätt som första instans.

Crafoordska stiftelsen

Emma Claesdotter-Knutsson
Lunds universitet
IKLV; Barn och ungdoms psykiatri
Baravägen 1
221 85 Lund

Lund 5/26/2020

20200862

Beslut, referensnummer 20200862

Vi har nöjet att meddela att vi beviljat 400,000 kr till ansökan med titeln: "Spel och spelproblem med och utan pengar i en barn och ungdoms population".

Bidraget får användas till de kostnader som specificerats i ansökan.

Läs villkoren för bidraget på baksidan av detta brev. Du bekräftar med inskickad rekvisition att du är införstådd med dem. I villkoren framgår när du kan rekvirera och vilka uppgifter vi behöver.

Bidraget betalas ut till universitetet/högskolan. Eventuell utrustning som inhandlas för bidraget ska registreras vid läroanstalten och blir dess egendom. Observera särskilt att bidraget inte får användas till din lön eller till stipendier.

Lycka till!

Crafoordska stiftelsen,

Leif Andersson, VD

FORTE: Forskningsrådet för hälsa, arbetsliv och välfärd

BESLUT
Beslutnr: STY-2021/0005
Beslutsdatum: 2021-09-30

BESLUT OM BIDRAG

Diarienummer: 2021-01696
Projektledare: Sabina Kapetanovic
Projekttitel: Betydelsen av föräldra-barnrelationen för ungdomars attityder och spel med eller utan pengar - kunskap och prevention

Beslutsinstans: STY - Styrelsebeslut
Handläggare: Andreas Björke

BESLUT
Forskningsrådet för hälsa, arbetsliv och välfärd har beslutat att ge bidrag enligt nedan:
2021-11-01 - 2021-12-31: 1 400 000 SEK
2022-01-01 - 2022-12-31: 1 200 000 SEK
2023-01-01 - 2023-12-31: 1 295 000 SEK
2024-01-01 - 2024-10-31: 0 SEK

Beslutet är fattat med stöd av 2 § förordningen (2007:1431) med instruktion för Forskningsrådet för hälsa, arbetsliv och välfärd (instruktion för Forte).

Enligt 14 § instruktion för Forte kan beslutet inte överklagas.

Enligt 11 § tredje stycket instruktion för Forte får Forte besluta att beviljade medel inte längre ska betalas ut, om den verksamhet som medlen avser inte uppfyller de krav som bör ställas. Beslutet får avse viss tid. Ett sådant beslut får överklagas till allmän förvaltningsdomstol.

Beslutet tilldelas
Högskolan Väst
Projektledare: Sabina Kapetanovic
